# A Novel Reproductive Mode in Frogs: A New Species of Fanged Frog with Internal Fertilization and Birth of Tadpoles

**DOI:** 10.1371/journal.pone.0115884

**Published:** 2014-12-31

**Authors:** Djoko T. Iskandar, Ben J. Evans, Jimmy A. McGuire

**Affiliations:** 1 School of Life Sciences and Technology, Institut Teknologi Bandung, Bandung, Indonesia; 2 Center for Environmental Genomics, Department of Biology, McMaster University, Hamilton, Ontario, Canada; 3 Museum of Vertebrate Zoology and Department of Integrative Biology, University of California, Berkeley, California, United States of America; Leibniz-Institute of Freshwater Ecology and Inland Fisheries, Germany

## Abstract

We describe a new species of fanged frog (*Limnonectes larvaepartus*) that is unique among anurans in having both internal fertilization and birth of tadpoles. The new species is endemic to Sulawesi Island, Indonesia. This is the fourth valid species of *Limnonectes* described from Sulawesi despite that the radiation includes at least 15 species and possibly many more. Fewer than a dozen of the 6455 species of frogs in the world are known to have internal fertilization, and of these, all but the new species either deposit fertilized eggs or give birth to froglets.

## Introduction

The Indonesian island of Sulawesi in central Indonesia is home to a largely unstudied radiation of fanged frogs in the genus *Limnonectes* Fitzinger, 1843 (Anura: Dicroglossidae Anderson, 1871). To date, only three species are recognized from Sulawesi, but at least 15 species occur on the island [1], [2], [3]. It was recently proposed that Sulawesi fanged frogs represent an adaptive radiation based on patterns of morphological diversification among co-distributed species, and correspondence of morphological features (body size, webbing) with habitat utilization [3]. Adult body size ranges from ∼2 g to more than 900 g in members of the Sulawesi assemblage, with large-bodied species found in close association with fast moving rivers, and small-bodied species occupying more terrestrial niches in forest leaf-litter and on the banks of slow moving streams. Iskandar and Tjan [4] first documented that there are multiple undescribed species of *Limnonectes* on Sulawesi, and indicated that one of the constituent species utilizes an unusual ovoviviparous reproductive strategy. Here we describe that species, and provide additional details on its reproductive mode.

Anurans exhibit tremendous diversity in mode of reproduction. Not only have there been many independent origins of direct development, the condition in which a free-living tadpole stage is bypassed and froglets emerge from the egg capsules [5], but a bewildering array of mechanisms has evolved wherein parents care for their developing offspring. Examples of parental care include guarding of terrestrial eggs, transport of tadpoles on the back following hatching, carrying developing eggs in pouches or depressions on the back and flanks, males carrying developing tadpoles in the vocal sac, and even the now-extinct species of gastric brooding frogs in which the female carried her developing tadpoles in her stomach (see [6] for a summary). Despite this extreme reproductive diversity, internal fertilization has evolved only a few times among the 6455 known species of anurans [7], [8]. Only the African bufonid genera *Nectophrynoides* and *Nimbaphrynoides* and the now extinct Puerto Rican species, *Eleutherodactylus jasperi*, are documented to combine internal fertilization with egg retention and subsequent birth of froglets [9]. To this group, we can now add a species of Sulawesi fanged frog in the genus *Limnonectes* with internal fertilization and live birth of tadpoles, a reproductive mode that is unique among anurans.

## Materials and Methods

We analyzed more than 100 specimens representing the new species from 23 localities on the island of Sulawesi. This study was conducted under an approved Institutional Animal Care and Use Committee protocol issued to JAM by the University of California at Berkeley (Protocol #R279). Fieldwork conducted in Indonesia was undertaken under research permits issued by LIPI and RISTEK, with specimen exportation authorized under permits or loans issued by LIPI, the Museum Zoologicum Bogoriense, and Pusat Studi Biologi. Specimens were captured by hand in the field, sacrificed via emersion in an aqueous solution of MS-222 (tricaine methane sulfonate) buffered to neutral pH, and prepared as formalin-fixed specimens deposited primarily in the Museum Zoologicum Bogoriense (the national museum of Indonesia) or Museum of Vertebrate Zoology at UC Berkeley. As part of a comprehensive study of Sulawesi *Limnonectes* diversification, we obtained morphometric data for the full Sulawesi assemblage. Sex was determined by inspection of gonads. The following measurements were made to the nearest 0.1 mm using digital calipers: snout-vent length (SVL) – from cloaca to tip of snout; head width (HW) – widest distance between posterior end of lower jaw; head length (HL) – from posterior end of lower jaw to tip of snout; femur length (FE) – from cloaca to distal end of femur; tibia length (TI) – from knee to distal end of tibia; foot length (FL) – from distal end of tibia-fibula to tip of 4th toe; inner metatarsal tubercle (IM); humerus length (UA) – from proximal end of upper arm to elbow; lower arm length (LA) – from elbow joint to base of middle palmar tubercle; hand length (HA) – from lower border of middle tubercle to distal tip of third finger; snout length (SL) – from bony border of eye socket to tip of snout; snout width at eye level (SWE); snout width at nostril level (SWN); eye diameter (EY) – outer diameter, measured from bones bordering eye; interorbital distance (IO) – distance between orbits ( =  width of frontoparietal bones); eye-tympanum distance (ET); eye-narial distance (EN) – eye socket border to posterior border of nostril; Internarial distance (IN) – shortest distance between nostrils; nostril to tip of snout (NT); tympanum diameter (TY); odontoid process length (OP) –length of fang-like odontoid process of lower jaw; tibia diameter measured at its widest point (TD). Digital webbing formulae from [10].

### Nomenclatural acts

The electronic edition of this article conforms to the requirements of the amended International Code of Zoological Nomenclature, and hence the new names contained herein are available under that Code from the electronic edition of this article. This published work and the nomenclatural acts it contains have been registered in ZooBank, the online registration system for the ICZN. The ZooBank LSID (Life Science Identifier) can be resolved and the associated information viewed through any standard web browser by appending the LSID to the prefix “http://zoobank.org/”. The LSID for this publication is: urn:lsid:zoobank.org:pub: A758E03A–C054–4193–9768–D0C4AD588AEA. The electronic edition of this work was published in a journal with an ISSN, and has been archived and is available from the following digital repositories: PubMed Central, LOCKSS.

## Results

### Taxonomy

**Figure 1:**
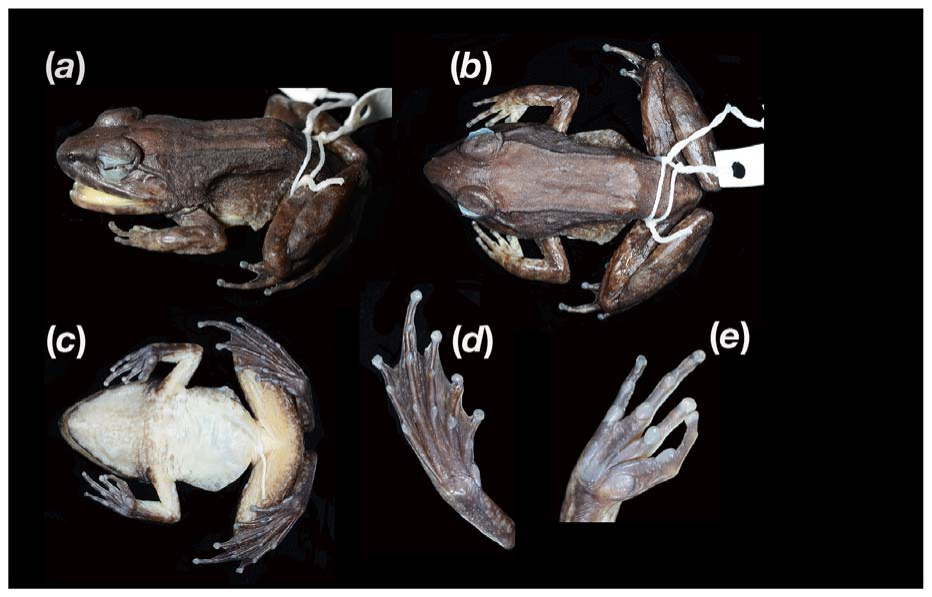
Images of the holotype of *Limnonectes larvaepartus* (MZB.Amph.23755) in (*a*) lateral, (*b*) dorsal, and (*c*) ventral view. Ventral views of the right foot (*d*) and right hand (*e*) are also presented.

**Figure 2:**
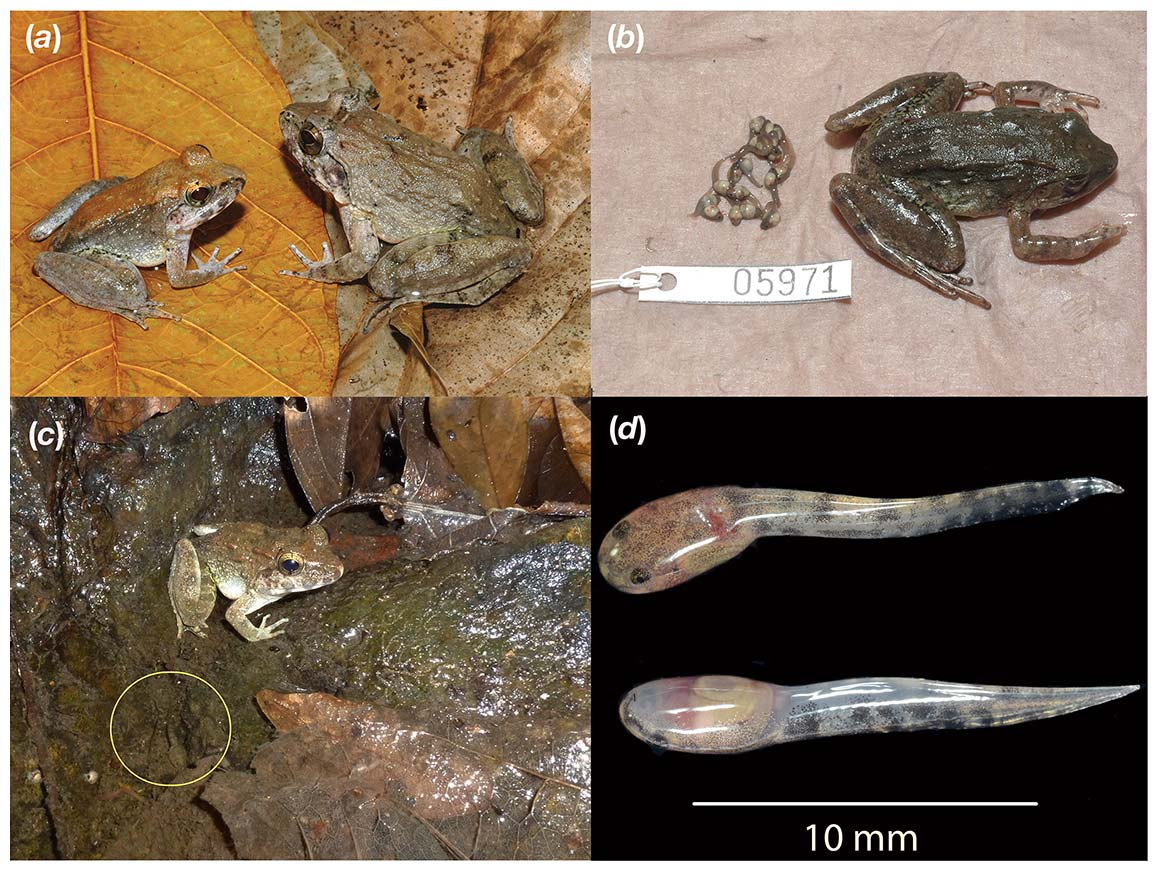
Images of *Limnonectes larvaepartus*. (*a*) MVZ 268323 (**male, left**) and MVZ 268307 (**female, right**) collected from Desa Uaemate along the Tasio-Tibo Road, Kabupatan Mamuju, Provinsi Sulawesi Barat, Sulawesi Island (02.61287S, 119.14238 E, 89 m elev.); (*b*) *Limnonectes larvaepartus* female (MVZ 268426) with tadpoles removed from the oviduct. Note the large yolk reserves available to the tadpoles; (*c*) An *in situ* adult male *L. larvaepartus* (JAM 14234) observed calling while perched on the edge of a small pool 2 m away from a 2 m wide stream; several *L. larvaepartus* tadpoles were present in the pool including the two visible within the yellow circle; (*d*) dorsal and ventral views of ∼stage 25 *L. larvaepartus* tadpoles (JAM 14271) released by a pregnant female (JAM 14237) at the moment of capture.

*Limnonectes* ***larvaepartus*** new species (Figs. 1, 2)

Urn:lsid:zoobank.org:act: 60AA7136-89A0-4DBB-9FBC-BD0FAF8A214C

This species has been referred to in the literature under the names *Limnonectes larviparus* [11] and *Limnonectes* “ovovivipar” [4], both of which created nomina nuda. This species also corresponds to *Limnonectes* sp. V in [2], [3].

### Etymology

The species name *larvaepartus* (from ‘larvae’, plural of larva, the early form of an animal, and ‘partus’, to give birth to) reflects the unique reproductive mode of this tadpole-bearing species.

### Holotype

MZB.Amph.23755 (Field Number: BSI 0605, see Fig. 1), an adult male, collected from Dunu Village, (0.92353^o^N; 122.64386^o^E) at 189 m elevation, Kecamatan Anggrek, Kabupaten Gorontalo, Provinsi Gorontalo, Sulawesi, Indonesia by J.A. McGuire & team, 18 October 2004.

### Paratypes

Paratypes (n = 30) are from Sulawesi Utara, Gorontalo, Sulawesi Tengah, and Sulawesi Barat Provinces: MZB 2834, a gravid female with 33 translucent tadpoles (Gosner stage 23) from the left oviduct and 2 from outside the body, from Toraut (00′33.72^o^N, 123′54.23^o^E), Bogani Nani Wartabone National Park, Kabupaten Bolaang Mongondow, Sulawesi Utara at 370 m elevation, by D. T. Iskandar, 15 August 1991; FMNH 252453, a female, from Toraut, Bogani Nani Wartabone National Park, Kabupaten Bolaang Mongondow, Sulawesi Utara by D. T. Iskandar, August 1991. MVZ 255545, 256009-11, 256013 from Desa Lombongo (−1.43346, 120.30800), Kecamatan Suwawa, Kabupaten Bone Bolango, Bogani Nani Warta Bone National Park, Provinsi Gorontalo at 75 m elevation by J. A McGuire and team, 20 October 2004. ZRC 1.3258 (left femur removed) from Toraut, Bogani Nani Wartabone National Park, Kabupaten Bolaang Mongondow, Sulawesi Utara at 370 m elevation, by D. T. Iskandar, 15 August 1991. MZB 2835–2841 from Toraut, Bogani Nani Wartabone National Park, Kabupaten Bolaang Mongondow, Sulawesi Utara at 370 m elevation, by D. T. Iskandar, 15 August 1991 & 12 July 1992. MVZ 255548–49 from Desa Pontak (−2.62910, 118.99300), Kecamatan Motoling, Kabupaten Minahasa Selatan, Provinsi Sulawesi Utara at 285 m elevation by J. A. McGuire and team, 13 October 2004. MZB 3117, near Potolok river, Lolak, Bogani Nani Wartabone National Park at 350 m elevation, Kabupaten Bolaang Mongondow, Sulawesi Utara; MZB 3118, male from seashore forest near Bungbungan River, Lolak, Bogani Nani Wartabone National Park, Kabupaten Bolaang Mongondow, Sulawesi Utara. MZB 3120 from Tangkorak River, Desa Pindol, Kecamatan Lolak, Bolaang Mongondow, Sulawesi Utara, by Mumpuni, 26 June 1995; MZB 3121 from Tangaga Forest, Dudepo, Bolaang Mongondow, Sulawesi Utara, by I. Maryanto, 20 October 1995; MZB 3122 from Potolok River, Bogani Nani National Park, Lolak, Sulawesi Utara by Mumpuni, 19 June 1995; MZB 3124 from Bungbungan River, Bogani Nani National Park, Lolak, Sulawesi Utara by Mumpuni, 19 June 1995; MZB Amph.8108 from Toraut, near Bogani Nani Wartabone National Park, Sulawesi Utara. LSUMZ 84209, 84214, 84221, 84224 from Desa Torout, Bogani Nani Wartabone National Park, Kabupaten Bolaang Mongondow, Provinsi Sulawesi Utara at 267 m elevation by J. A. McGuire on 6 and 11 September 2001.

### Other referred specimens

LSUMZ 84218, 84219 from Desa Torout, Bogani Nani Wartabone National Park, Kabupaten Bolaang Mongondow, Provinsi Sulawesi Utara by J.A. McGuire. FMNH 130991, 106930 from Buang-buang Island, Sulawesi Utara. MZB 3119, juvenile from Seashore forest near Bungbungan River, Lolak, Bogani Nani Wartabone National Park, Kabupaten Bolaang Mongondow, Provinsi Sulawesi Utara. AMNH 167199 from Tangkoko National Park (1.570083^o^N, 125.156933^o^E), Kabupaten Minahasa, Provinsi Sulawesi Utara. MVZ 255546 from Desa Salumpaku (−1.60757, 119.29900), Kecamatan Banawa, Kabupaten Donggala, Provinsi Sulawesi Tengah. MVZ 255547 from Desa Kelapa Dua (−1.60757, 119.29900), Kecamatan Anreapi, Kabupaten Polewali Mandar, Provinsi Sulawesi Barat. MVZ 268426, 268428–30, 268432 from Polewali-Masawa Road (River 1) (−2.65490, 118.93300), Kecamatan Polewali, Kabupaten Polewali Mandar, Provinsi Sulawesi Barat. MVZ 268309–10, 268313, 268317–19, 268322, 268325, MZB.Amph. 20675, 20677–80 from Desa Uaemate (Tasiu-Tibo Road; S02.61550, E119.14417), Kecamatan Kaluku, Kabupaten Mamuju, Provinsi Sulawesi Barat. MZB.Amph.20663 from Desa Kabiraan (−2.62460, 119.14700), Kecamatan Ulumunda, Kabupaten Majene, Provinsi Sulawesi Barat.

### Distribution

**Figure 3:**
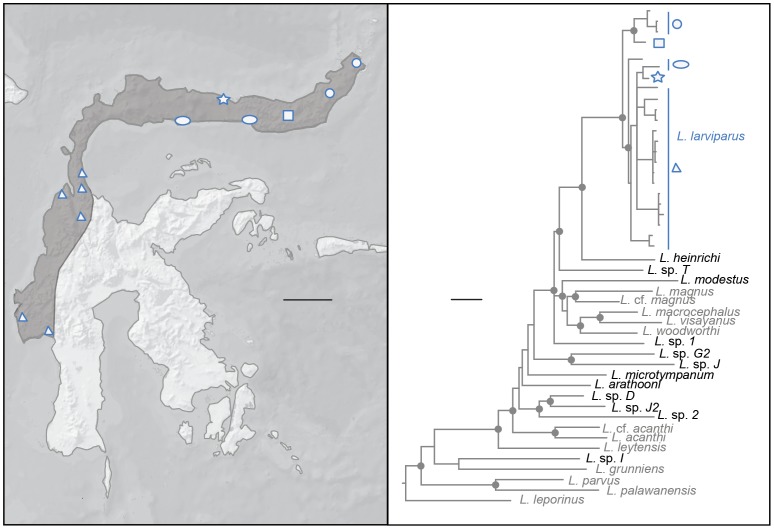
Distribution map (left panel) depicting the range of *Limnonectes larvaepartus*. The right panel shows the phylogenetic position of *L. larvaepartus*, with different symbol shapes denoting regional genetic structure in the species.

*Limnonectes larvaepartus* occurs across the Northern Peninsula, as well as on the western margin of Sulawesi's Central Core (Fig. 3). We do not know the full extent of the species' range in the Central Core because the central highlands of Sulawesi remain poorly explored herpetologically. Several genera have species range boundaries in this same general region (e.g. the flying lizards *Draco spilonotus* and *D. walker* [12], the fanged frogs *Limnonectes* sp. *I* and *L.* sp. *D* [3], and the tarsiers *Tarsius lariang* and *T. dentatus* [13].

### Diagnosis

Prior workers have recognized substantial species diversity in the genus *Limnonectes* on Sulawesi. However, diagnosing many of these lineages on the basis of morphology is challenging, and several authors have instead opted to apply names to Sulawesi specimens representing species from outside Sulawesi. Consequently, the following names have all been incorrectly applied to Sulawesi populations: the Lesser Sundas species (type locality: Flores Island) *L. dammermani* (Mertens, 1927), the Bornean species *L. finchii* (Inger, 1966), the Mollucan species (type locality: Ambon) *L. grunniens* (Daudin, 1801), and the Philippine taxa *L. leytensis* (Boettger, 1893), *L. magnus* (Stejneger, 1909), and *L. palavanensis* (Boulenger, 1894). These names should not be applied to Sulawesi populations, as was verified phylogenetically for several of these taxa [3]. Only four Sulawesi species have been described: *L. arathooni* (Smith, 1927), *L. heinrichi* (Ahl, 1933), *L. modestus* (Boulenger, 1882), and *L. microtympanum* (van Kampen, 1909). However, we will show elsewhere that *L. heinrichi* is a junior synonym of *L. modestus* and the species complex that we have referred to previously [2], [3] as *L. modestus* remains undescribed – thus, at present there are but three valid described species of Sulawesi *Limnonectes*.

*Limnonectes larvaepartus* can be distinguished from all other described species of *Limnonectes* by its reproductive mode (Fig. 2). It can be further differentiated from all described Sulawesi species by its combination of body size (mean male SVL = 37.4; female SVL = 40.2 mm), coloration, tympanum size, and degree of hind foot webbing, as well as on the basis of phylogenetic placement (Fig. 3). *Limnonectes arathooni* is endemic to Sulawesi's Southwestern (SW) Peninsula south of the Tempe Depression, and thus does not occur within the range of *L. larvaepartus*. It is similar in size to the new species (male SVL = 36.6 mm, females = 39.6 mm), but differs in having substantially reduced webbing (extending to penultimate phalange of fourth toe vs. to toe disc), in lacking fine granular dorsal tubercles, and in having melanic spots above the forelimb insertion, a fine ridge extending posteriorly behind each eye, and an alternative derived reproductive mode in which males guard clutches of terrestrial eggs that hatch into tadpoles that then make their own way to an adjacent stream by sliding down steep stream-side embankments [14]. *Limnonectes microtympanum*, like *L. arathooni*, is restricted to the SW Peninsula south of the Tempe Depression, and thus does not overlap in geographical range with *L. larvaepartus*. *Limnonectes microtympanum* is moderately large (male SVL = 78.4; female SVL = 72.4 mm), and thus much larger than the new species. *Limnonectes microtympanum* also differs from the new species in having proportionally smaller tympana (TY/SVL = 0.05+0.01 in males, 0.06+0.01 in females versus 0.08+0.01 in both sexes in *L. larvaepartus*). The new species occurs in broad sympatry with *L. modestus*, which is a moderate sized (male SVL = 70.2 mm; female SVL = 64.0 mm) inhabitant of fast-moving streams and substantially larger than *L. larvaepartus*. Like *L. larvaepartus*, *L. modestus* has nearly complete hindfoot webbing (slightly more extensive in *L. modestus* than in *L. larvaepartus* but reaching the toe disc in both species), a dusky throat with melanic pigments extending onto the pectoral region (in a clear wedge shape in *L. modestus*, more randomly distributed in *L. larvaepartus*), and skin with extensive fine granular tubercles. *Limnonectes modestus* also exhibits a derived reproductive mode involving production of a relatively small number of large (10 mm diameter) eggs that are deposited along the edge of fast moving streams.

### Description of the holotype

An adult male (Fig. 1) 48 mm SVL, body moderately robust, head not broader than body, head about 65% longer than wide, length 45% of snout-vent length, snout 17% of snout-vent length, moderately pointed, projecting above lower jaw, nostril lateral, closer to tip of snout than to eye, lore essentially straight, canthus rostralis distinct, eye about equal to snout length, pupil diamond-shaped, upper eyelid with tubercles; interorbital region smooth, width 69% of internarial distance, tympanum moderate, slightly wider than interorbital distance, supratympanic fold distinct, extending from posterior corner of eye to supra-axillary region, in contact with tympanic annulus, temporal muscle slightly enlarged; odontoid process 2.1 mm. Dentigerous process of vomer distinct, angled anterolaterally, approximately at 45^o^ angle, posterior ends separated by distance approximately equal to one-third diameter of choanae. Limbs relatively slender, tibia width at thickest part 7.5 mm; femur length 52% of snout-vent length, heels moderately overlapping when placed perpendicular to body axis; tibia length 64.9% of foot length, 48.9% of snout-vent length; foot length 71% of snout-vent length; tarsal fold indistinct, only evident as a ridge; toe discs moderately expanded, circum-marginal groove horseshoe-shaped, pointed anteriorly. Plantar surface of foot smooth, subarticular tubercle rounded, relative length of toes 4>3>5>2>1. Inner metatarsal tubercle prominent, elongate, ovoid with a sharp spade like ventral edge; outer metatarsal tubercle absent; hind foot webbing full, extending to toe discs, slightly emarginated. Manus length 51.3% of foot length, fingers slender, terminal discs slightly expanded, length formula 3>1≥4≥2, with slight differences in length, subarticular tubercle rounded, convex; supernumerary tubercles absent; inner and outer metacarpal tubercles enlarged, nuptial pads and webbing absent, forearm muscle not enlarged. See Table 1 for measurements and variation.

**Table 1:** Summary of univariate morphological variation among mensural characters in *Limnonectes larvaepartus*.

	*Limnonectes larvaepartus*		
	Males (31)	Females (30)	Holotype
Snout-Vent Length (SVL)	39.8+4.7	41.2+3.1	48.0
Range	31.3−48.3	35.7−47.5	-
Head Length (HL)	17.6+2.0	17.9+1.3	21.5
Head Width (HW)	15.4+2.1	15.1+1.5	18.9
Snout Length (SL)	7.0+0.9	7.2+0.5	8.2
Eye-Narial Distance (EN)	3.6+0.5	3.9+0.4	4.0
Nostril-Tip of Snout (NT)	3.6+0.4	3.8+0.4	4.3
Internarial Distance (IN)	4.5+0.6	4.4+0.4	4.9
Snout Width at Eye (SWE)	10.5+1.2	10.6+1.0	12.2
Snout Width at Nostril (SWN)	6.0+0.8	5.8+0.7	7.5
Eye-Tympanum Distance (ET)	1.8+0.5	1.6+0.4	1.7
Odontoid Process Length (OP)	2.0+0.3	1.6+0.2	2.1
Tympanum Diameter (TY)	3.1+0.5	3.3+0.4	3.6
Interorbital Distance (IO)	3.0+0.5	3.0+0.4	3.9
Eye Diameter (EY)	7.2+0.8	7.4+0.5	8.7
Femoral Length (FE)	21.1+2.6	22.0+2.4	23.7
Tibial Length (TI)	22.7+2.8	24.1+2.3	23.5
Foot Length (FL)	32.1+3.6	33.5+3.0	36.2
Humeral Length (UA)	11.5+1.0	11.4+1.1	13.5
Lower Arm Length (LA)	8.4+1.0	9.1+1.0	9.5
Hand Length (HA)	9.7+1.2	9.9+1.0	11.8
HL/SVL	0.44+0.02	0.44+0.03	0.45
HW/SVL	0.39+0.02	0.37+0.02	0.39
SL/SVL	0.18+0.01	0.18+0.01	0.17
EN/SVL	0.09+0.01	0.09+0.01	0.08
IN/SVL	0.11+0.01	0.11+0.01	0.10
SWE/SVL	0.26+0.02	0.26+0.01	0.25
SWN/SVL	0.15+0.01	0.14+0.01	0.16
ET/SVL	0.04+0.01	0.04+0.01	0.04
OP/SVL	0.05+0.01	0.04+0.01	0.04
TY/SVL	0.08+0.01	0.08+0.01	0.08
IO/SVL	0.08+0.01	0.07+0.01	0.08
EY/SVL	0.18+0.01	0.18+0.01	0.18
FE/SVL	0.53+0.03	0.53+0.03	0.49
TI/SVL	0.57+0.04	0.59+0.04	0.49
PL/SVL	0.81+0.03	0.81+0.04	0.75
SL/SWE	0.67+0.07	0.69+0.07	0.67
IN/SWN	0.74+0.08	0.76+0.08	0.65
HW/HL	0.87+0.07	0.84+0.08	0.88
SL/HW	0.46+0.05	0.48+0.03	0.43
IO/IN	0.68+0.07	0.67+0.08	0.80
TI/TD	3.6+0.4	3.8+0.2	3.6

### Coloration

The dorsal coloration is highly variable, typically brownish-grey, but can be darker brown on the dorsolateral region, and some individuals are reddish-brown or golden-tan (see Fig. 2). ∼23% of specimens have a bold mid-dorsal stripe. The venter is either yellowish or cream colored, with the upper end of the tibia usually bearing a prominent dark spot. A light bar is often present in the interorbital region, and the coloration of the snout to interorbital region may be lighter than the remainder of the dorsum. The tympanum is often masked in black leaving only the lower rim sharing the predominant body coloration. The gular region is usually darker in males and may have a finely mottled wedge-shaped melanic patch. The dorsal half of the iris is golden-orange in coloration in at least some individuals (we do not know of exceptions, but have not documented iris coloration for most specimens).

### Eggs and tadpoles

Females produce ∼100 non-pigmented eggs (see [15]), though the most we have observed is 55, which possibly represents the contents of just one oviduct. The eggs lack a jelly-coat and reach at least ∼3 mm in diameter. These eggs develop within the oviducts into pigmented tadpoles that reach at least Gosner stage 35 prior to parturition (see [16] for staging). The gut is initially provisioned with substantial yolk (Fig. 2*b*), and developing tadpoles prematurely removed from the oviducts at approximately stage 21 progressed to approximately stage 25 over the course of two weeks in a water bottle without supplemental food, suggesting plasticity in terms of the timing of parturition. A detailed description of the tadpole is provided in the accompanying paper by Kusrini et al. [15].

### Natural history

*Limnonectes larvaepartus* occurs in natural and disturbed forest habitats of Sulawesi, generally in sympatry with at least one, and sometimes as many as five other *Limnonectes* species. In the western Central Core of Sulawesi, we have always found *L. larvaepartus* living in sympatry with a much larger species that has been referred to in the literature as *L.* sp. *D* (2,3). Whereas *L.* sp. *D* is generally found on rocks in fast-moving streams or within a meter of water on the banks of fast-flowing streams, *L. larvaepartus* is usually found further from the stream (2–10 meters from water) on rocky substrates, in leaf-litter, or secluded in grassy vegetation. Because we have observed that *L.* sp. *D* predates other frogs, including other *Limonectes*, it is possible that *L. larvaepartus* avoids large streams in response to predation pressure from larger *Limnonectes* species. Male *L. larvaepartus* typically call from the margins of seeps, puddles, or small pools away from the main stream. Notably, we have found many males calling from small pools that were already inhabited by *L. larvaepartus* tadpoles (Fig. 2*c*), with as many as three size-classes of tadpoles represented. It is unclear whether some or all of the observed tadpoles were sired by the accompanying male. We have furthermore collected at least one pregnant female from a small stream-side puddle already inhabited by tadpoles of two size classes, again suggesting the possibility that individual pools may be visited repeatedly by the same adult males and females during the reproductive season.

## Discussion

We first became aware of the unusual reproductive mode of *Limnonectes larvaepartus* while conducting fieldwork on Sulawesi. In several instances, we discovered tadpoles in the oviducts while preparing specimens. In each case, having sacrificed frogs for preparation, the abdominal wall was observed to quiver, and incision resulted in living tadpoles emerging from the opening (see S1 Movie). On one occasion, a gravid female gave birth to tadpoles in-hand at the moment of capture. On four other occasions, field-collected *L. larvaepartus* in our possession gave birth to tadpoles while being held individually in collecting bags. In total, we have either observed tadpoles in the oviducts or direct-birth of tadpoles on 19 occasions.

Because we have not witnessed natural birth of tadpoles in free-living frogs, two possible alternative reproductive modes are possible for this species. *Limnonectes larvaepartus* reproduction may simply reflect what we have observed in the hand – direct birth of tadpoles. Alternatively, this species may be capable of retaining developing young in the oviducts through metamorphosis with subsequent birth of froglets, as is the case for *Eleutherodactylus jasperi* and members of the African bufonid genera *Nectophrynoides* and *Nimbaphrynoides* [9], [17], [18]. The latter mode seems unlikely for several reasons. First, we have collected 19 pregnant individuals carrying tadpoles in the oviducts but none carrying froglets. These 19 females were collected across different months, years, and localities. Second, we have observed and collected at least four clutches of free-living *L. larvaepartus* tadpoles in small pools of water on the margins of streams, with each of these samples exhibiting two or three size classes and thus possibly representing multiple clutches. Three of these clutches were accompanied by males, some of which were calling, and one was found in association with the gravid female that gave birth to 55 tadpoles (some in the hand of JAM, others subsequently deposited in a collecting bag). Finally, although adaptive plasticity has been demonstrated for many frog species, whereby tadpoles or froglets are capable of hatching from their eggs prematurely when attacked by predators [19], this has only been documented for a single direct-developer, *Eleutherodactylus coqui* [20]. In the case of *E. coqui*, the capacity for early hatching commenced at stages 13 or 14, whereas normal hatching occurred at stage 15 (see [21] for staging). Thus, *E. coqui* were capable of premature hatching only as froglets, with toe pads and eyelids present but before the tail was completely resorbed, a stage much later than would be required by *L. larvaepartus* if it were simultaneously capable of hatching as either a tadpole or full-term froglet. Most direct-developers, as well as the few frog species that give birth to froglets, pass through the tadpole stage in a form poorly suited for free-living. In many such species, the tadpole has a broad, highly vascularized ‘respiratory tail’ specialized for intra-egg or intra-oviductal gas exchange rather than for swimming, as is the case for *Eleutherodactylus jasperi* [17], and may lack mouthparts and functional gills [22], [23], [24]. In others, the tail remains rudimentary and limb buds appear early in development such that a typical tadpole phenotype never occurs [22], [23]. Given these observations, it appears unlikely that any one species would be characterized by the combination of (1) internal fertilization, (2) complete metamorphosis within the oviducts and live-birth in the form of froglets, as well as (3) an oviductal tadpole stage that is capable of premature birth and free-living. Although we think that *L. larvaepartus* is much more likely to give birth to tadpoles as its sole mode of reproduction as opposed to exhibiting adaptive plasticity allowing for birth of either tadpoles or froglets depending on the circumstances confronting the frog, we note that either condition would be unique among Anura. In either case, *L. larvaepartus* requires internal fertilization, which is, itself, extremely rare among anurans [8], [25]. The mechanism by which internal fertilization takes place is unknown, and there is no obvious intromittent organ present to facilitate sperm transfer. If *L. larvaepartus* reproduction always involves birth of tadpoles, it is likely that they are ovoviparous given that the tadpoles are well provisioned with yolk and appear fully capable of developing without nutrient transfer from the mother. Prior to parturition, the tadpoles have well-developed tails and pigmentation, and well developed mouthparts. Given this morphology, it is unlikely that these tadpoles are endotrophic (never feed before metamorphosing into froglets). It is more likely that the tadpoles are born after exhausting their yolk supply, and are subsequently self-feeding prior to metamorphosis. Nevertheless, it is clear that much remains to be learned about this unusual frog, the discovery of which brings to light yet another axis of diversity characterizing the remarkable Sulawesi fanged frog adaptive radiation.

## Supporting Information

S1 Movie **Video showing the characteristic quivering abdomen caused by movement of tadpoles within a pregnant female** ***Limnonectes larvaepartus.***
(MOV)
