# Correction: A Novel Reproductive Mode in Frogs: A New Species of Fanged Frog with Internal Fertilization and Birth of Tadpoles

**DOI:** 10.1371/journal.pone.0119988

**Published:** 2015-03-16

**Authors:** The PLOS ONE Staff

There is an error in the fifth sentence of the second paragraph within the Diagnosis sub-section of the Results section. The correct sentence is: *Limnonectes microtympanum* is moderately large (male SVL = 78.4; female SVL = 72.4 mm), and thus much larger than the new species. *Limnonectes microtympanum* also differs from the new species in having proportionally smaller tympana (TY/SVL = 0.05±0.01 in males, 0.06±0.01 in females versus 0.08±0.01 in both sexes in *L. larvaepartus*). The publisher apologizes for the error.

## References

[1] Iskandar DT, Evans BJ, McGuire JA (2014) A Novel Reproductive Mode in Frogs: A New Species of Fanged Frog with Internal Fertilization and Birth of Tadpoles. PLoS ONE 9(12): e115884 doi: 10.1371/journal.pone.0115884 25551466 10.1371/journal.pone.0115884 PMC4281041

